# Dynamic Mechanical Properties and Energy Absorption Capabilities of Polyureas Through Experiments and Molecular Dynamic Simulation

**DOI:** 10.3390/polym17010107

**Published:** 2025-01-02

**Authors:** Ke Yang, Shanda Wang, Yanru Chen, Hanhai Dong, Quanguo Wang, Qingli Cheng

**Affiliations:** 1 State Key Laboratory of Chemical Safety, Qingdao 266000, Chinadonghh.qday@sinopec.com (H.D.);; 2 Sinopec Research Institute of Safety and Engineering Co., Ltd., Qingdao 266000, China

**Keywords:** polyurea, mechanical property, molecular dynamic simulation, energy absorption capabilities

## Abstract

Polyurea (PUR) has been widely used as a protective coating in recent years. In order to complete the understanding of the relationship between PUR microstructure and its energy absorption capabilities, the mechanical and dynamic performance of PURs containing various macrodiol structural units were compared using material characterization techniques and molecular dynamic simulation. The results showed that the PUR polycarbonate diols formed as energy absorbing materials showed high tensile strength, high toughness, and excellent loss factor distribution based on the comparison of stress–strain tensile curves, glass transition temperatures, phase images, and dynamic storage loss modulus. External energy from simple shear deformation was absorbed to convert non-bond energy, in particular, based on fractional free volume, interaction energy, and total energy and hydrogen bond number change from the molecular dynamic simulation. Hydrogen bonds formed between soft segments and hard segments in the PURs have been proven to play a significant role in determining their mechanical and dynamic performance. The mechanical and dynamic properties of PURs characterized and tested using experimental techniques were quantified effectively using molecular dynamic simulation. This is believed to be an innovative theoretical guidance for the structural design of PURs at the molecular level for the optimization of energy absorption capabilities.

## 1. Introduction

The number of the types and outputs of dangerous chemicals are progressively increasing with the rapid growth of the chemical industry all over the world, which leads to a dramatic increase in fire and explosion accidents [1,2,3,4]. The United Nations International Labor Organization stated that 2.78 million people die every year from chemical-related occupational diseases and accidents. The cost of these diseases and accidents is nearly USD 2.9 trillion [5]. In order to reduce the damage caused by chemical explosions, the protective materials with high strength and high toughness are extensively used in buildings, vehicles, and storage tanks [6,7,8,9].

In recent years, polyurea (PUR) has been widely used as a protective coating for bulletproofing and reducing shock waves because of its excellent dynamic and high-mechanical properties [10,11,12,13]. The dynamic mechanical properties and energy absorption mechanism of PUR have attracted researchers’ attention [14,15,16]. This is because PUR shows high-strain-hardening phenomenon under the high-speed load condition, and the dynamic mechanical response of the chain section to the glass state is distinct from that of rubber accompanied by a large amount of energy dissipation [17,18]. In addition, PUR behaves like block copolymers: the soft phase of PUR leads to rapid deformation to form a smaller and obscure microphase structure with a tensile deformation increased, while the hard phase of PUR rotated to form new microphase structures along the stretching direction [19,20,21]. Moreover, the tensile deformation of PUR could cause hydrogen bond cleavage and rearrangement in its hard phase, leading to energy absorption and dispersion [22,23,24,25].

Microstructures of polymers, such as chemical composition [26,27,28,29,30], molecular chain length [31,32], crystallization [33,34], and microphase separation [35,36,37,38], can affect the mechanical and dynamic properties of PUR. Although there are many studies on the dynamic properties and energy absorption of PUR, there are few discussions on the quantitative relationship of the microstructure of PUR and the dynamic mechanical properties of energy absorption [11,39]. Molecular dynamic simulation is a powerful tool to establish a direct quantitative relationship between microstructure and macroscopic properties, and it could also allow for the observation of microstructure changes in materials and the prediction of material properties [40,41,42,43,44,45].

Our team has also conducted detailed research on the resistance of polyurea to hydrocarbon explosions [46,47]. To further enhance the explosion resistance of polyurea materials, it is imperative to conduct an in-depth investigation into the energy absorption mechanisms of polyurea materials. In this article, the mechanical and dynamic properties of various PURs with four typical macrodiol structural units were investigated using characterizations and tests, as well as molecular dynamic simulation (MD). The performances of PURs with various macrodiol structural units were quantified effectively at the molecular level to reveal an energy absorption mechanism with the combination of experimental results and simulation data.

## 2. Materials and Methods

### 2.1. Materials

Polycarbonate diol (PC1000, *M_n_* = 1000) from Covestro, Polycaprolactone diol (PCL1000, *M_n_*= 1000), Poly(propylene glycol) (PPG1000, *M_n_* = 1000), and Polytetramethylene ether glycol (PTMG1000, *M_n_*=1000) from Aladdin Bio-Chem Technology were all dried for 2 h under vacuum at 100 °C and then stored in N_2_ atmosphere. Poly(propylene glycol) bis(2-aminopropyl ether) (D2000, *M_n_* = 2000), Poly(propylene glycol) bis(2-aminopropyl ether) (D230, *M_n_* = 230), and Isophorone diisocyanate (IPDI) were purchased from Aladdin Bio-Chem Technology, used as received. N, N-Dimethylformamide (DMF) was dried using 3-Åmolecular sieves.

### 2.2. PUR Synthesis and Film Preparation

The four kinds of polymerization reactions to form PURs between four kinds of diols (PC1000, PCL1000, PPG1000, or PTMG1000) and IPDI were conducted in a glove box under an argon atmosphere. These four PURs were named using the abbreviation of the corresponding diol. A typical polymerization process of PPG1000-PUR is shown as follows. IPDI (5.55 g, 25 mmol) was first dissolved in DMF (5 mL) in a 100 mL glass flask, and then PPG1000 (5.00 g, 5 mmol) was added into the flask. The whole solution was stirred for 4 h at 80 °C to form a pre-polymer, and then the solution was cooled down to room temperature. Another 10 g of DMF was then added to the solution to adjust its viscosity. Finally, the pre-polymer was added to the mixture of D2000 (9.00 g, 4.5 mmol) and D230 (3.56 g, 15.5 mmol) at room temperature for a further stirring of 2 h to form PPG1000-PUR.

To prepare samples for characterization and tests, the PPG1000-PUR solution was poured into a Teflon square mold (150 mm × 150 mm × 1 mm), and then gradually heated from 25 °C to 100 °C for 48 h. Residual solvent was removed under vacuum at 80 °C for 24 h. The thickness of the four PURs were within the range of 0.6 to 0.8 mm. Based on the SEM of cross sections, no bubble was observed inside the samples (Figure S1).

### 2.3. Characterization and Measurements

Molecular weights were measured using a Gel Permeation Chromatography (GPC, RI 2414, Waters, Milford, MA, USA) at a flow rate of 1.0 mL/min in THF at 35 °C with polystyrene calibration and a Multi-Angle Laser Light Scattering (SLS, Wyatt, Santa Barbara, CA, USA) at a flow rate of 1.0 mL/min in THF at 35 °C. A Fourier-transform Infrared Spectroscopy (FTIR, Nicolet 6700, Thermo Fisher Scientific, Waltham, MA, USA) fitted with a diamond ATR cell was used to obtain infrared spectra of the absorption of the materials. Optical photos were collected with polarized light using a Polarizing Microscope (POM, S60, Hui Tong Optical Instrument Co. Ltd., Shanghai, China) at room temperature. A X-ray Powder Diffraction (XRD, D8, Bruker, Karlsruhe, Germany) with a copper-point-focused source (λ = 0.154 nm) at 50 kV was used for phase identification of the crystalline material and provided information on interdomain spacings. Micron morphology was characterized using a Scanning Electron Microscope (SEM, S3400, Hitachi, Japan). A Atomic Force Microscope (AFM, SPM9700, SHIMADZU, Kyoto, Japan) in tapping mode was used to collect phase images. Thermal properties were characterized using a Differential Scanning Calorimeter (DSC, Q2000, TA, Santa Fe Springs, CA, USA) and a Thermal Gravimetric Analyzer (TGA, Q500, TA, Santa Fe Springs, CA, USA) under nitrogen with the heating rate of 10 °C min^−1^. Mechanical performance was tested using an Universal Testing Machine (Labthink 1510, Jinan, China) in tensile test mode at 100 mm/min. Dynamics were tested using a Dynamic Mechanical Analyzer (DMA, Q800, TA, Santa Fe Springs, CA, USA), including loss factor (tan δ), storage modulus, and loss modulus, in tension mode with a temperature range of −100–80 °C and a frequency range of 0–100 Hz.

### 2.4. Computational Section

All data obtained in this paper are from Materials studio (2020, BIOVIA, San Diego, CA, USA). The COMPASS force field was chosen for the analog modules Forcite and Amorohous Cell and the Condensed-Phase Optimized Molecular Potential for atomistic simulation studies [44]. PUR chains were created from PPG1000, PTMG1000, PCL1000, PC1000, IPDI, D2000, and D230 molecules with the ratios provided by Build module. The potential energy minimization of the PUR chains within the Forcite module was carried out at 298 K using Smart algorithms (combining the Steepest Decent, Conjugate Gradient, and the Newton’s minimization algorithms). PUR simulation boxes were created using Amorphous Cell module and were annealed from 298 K to 598 K for 5 cycles to relax polymer chains. MD simulation was conducted for 1000 ps at NPT condition (0.1 Mpa and 298 K) until the box size became constant, and then NVT condition (constant size and 298 K) was used for 1000 ps and equilibrium was reached. Finally, the computation cell of size was 3.4 nm × 3.4 nm × 3.4 nm, and the cutoff radius is 1.55 nm [48]. All of the computation results were the average value from 100 MD simulation frames.

These model errors were revealed to be lower than 2% when compared to Density_MD_ from the MD simulation and Density_Exp_ from the experiments (Table 1).

## 3. Results

### 3.1. Polymerization Result Analysis

**Figure 1:**
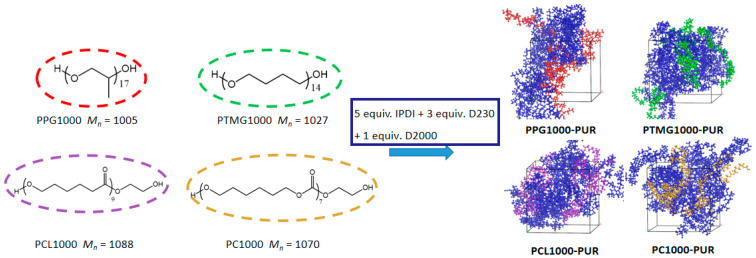
Schematic photo of synthetic routes and molecular models of the four types of PURs.

The synthetic routes of the PURs are shown in Figure 1. A series of macrodiols including PPG1000, PTMG1000, PCL1000, and PC1000 were reacted with the given IPDI-DMF solution to synthesize the pre-polymer solution. Then, D2000 and D230 were added into the pre-polymer solution to form the four PURs as chain extenders. PPG1000-PUR and PTMG1000-PUR are polyether-type PURs, while PCL1000-PUR and PC1000-PUR are polyester-type PURs. Because the high reactivity of the amine group and isocyanate group bring uncontrollable polymerization, the pre-polymerization of diisocyanate and macrodiols as capping agents is necessary to control the synthesis of PUR. In an effort to gain further insight into the effect of the macrodiol structural units on material properties, the macrodiol structural units in the PURs were considered as the macrodiol segments, and the other structural units including IPDI, D2000, and D230 were considered as the resin segments. The macrodiol segments of the four PURs still remained at 24 wt% by controlling feed ratio. Detailed results after polymerization are shown in Table 2. The molecular weights (*M_n_*) of each of the four PURs were quite similar, and they all had a unimodal and relatively narrow molecular weight distribution (MWD) in the GPC curves (Figure S2). This indicates that no gelation and other side reactions were carried out during polymerization; hence, the four PURs all had comparable molecular weight and topological structure.

### 3.2. Thermal Property Analysis

The thermal properties of all the resulting PURs were characterized using DSC. In the first DSC heating scan of heat history (Figure S3), no clear thermal transition behavior of PURs was observed. In the second heating scan (Figure S4), the four PURs had similar macrodiol segments T_g DSC_ about −60 °C, and no endothermic or exothermic transitions aside from T_g DSC_ are evident in these PURs, which suggests there is no typical thermal transition and T_m_s in these PURs. Based on TGA curves (Figure S5), PPG1000-PUR had a relatively low thermal decomposition temperature (T_d_ = 243 °C), and the T_d_s of other PURs was approximately 270–290 °C.

### 3.3. Microscopic Morphology Analysis

**Figure 2:**
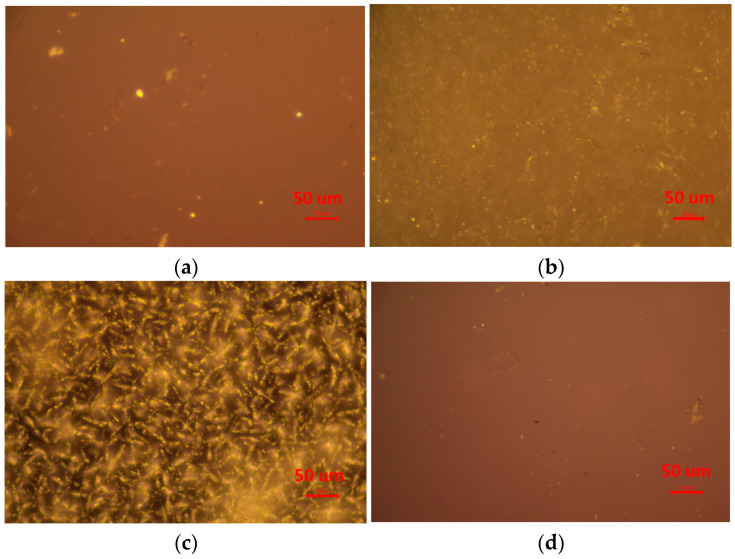
Optical photos of the four PURs: (**a**) PPG1000-PUR, (**b**) PTMG1000-PUR, (**c**) PCL1000-PUR, (**d**) PC1000-PUR.

The POM photos of the four PURs are presented in Figure 2. On the one hand, no clear crystallizing phase was supposed to be found in the POM photos of PPG1000-PUR or PC1000-PUR. On the other hand, the crystallizing phase were observed in the POM photos of PTMG1000-PUR and PCL1000-PUR, which synthesized because of semi-crystalline PTMG1000 and PCL1000. Especially, PCL1000-PUR showed the typical extinction of crystalline. This indicates that the semi-crystalline macrodiol soft segment could form a crystallizing phase in the PURs. Moreover, in the XRD profile of PCL1000-PUR, there was a sharp peak at 21.68°, which proved that the crystallizing phase existed in the PCL1000-PUR (Figure S6). However, the T_m_s of PTMG1000-PUR and PCL1000-PUR were not detected in the DSC curves (Figure S3), probably because of low crystallizing degree.

**Figure 3:**
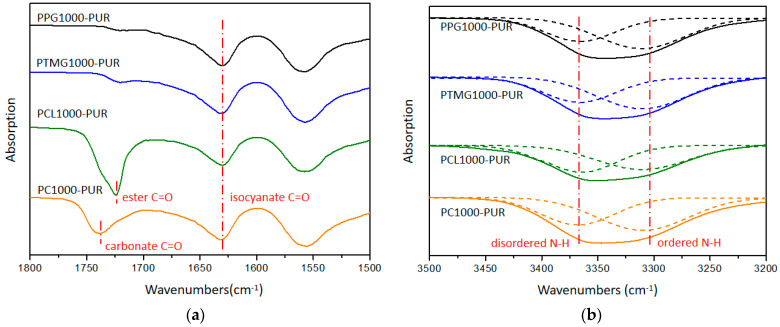
ATR-FTIR spectra of the PURs: (**a**) attribution of C=O peaks, (**b**) state of N-H association.

ATR-FTIR spectra of all PURs showed absorption peaks at 1630 cm^−1^ that corresponded to the C=O bond of isocyanate groups, and PCL1000-PUR and PC1000-PUR also featured the two special peaks at 1724 cm^−1^ and 1738 cm^−1^, which corresponded to the C=O bond of ester groups of caprolactone and carbonate, respectively (Figure 3a and Figure S7). Moreover, N−H stretching regions in the ATR-FTIR of PUR could be used to infer H-bond association in PUR [32]. The N−H bond assignments are 3310 cm^−1^ and 3368 cm^−1^ for ordered and disordered N−H groups, respectively. The wide N−H stretching peaks in the ATR-FTIR of PUR were separated into two special peaks for ordered and disordered N−H groups by Gauss fitting (Figure 3b). Based on the two peaks’ area ratio (ordered/disordered), the H-bond association values of PUR increased in the order PCL1000-PUR (1.06) < PTMG1000-PUR (1.57) < PPG1000-PUR (1.69) < PC1000-PUR (1.71). Perhaps because the soft segment of PCL1000-PUR was crystallized to restrict the H-bond formation, the H-bond association values of PCL1000-PUR were relatively low.

Tapping-mode AFM-phase images showed the microphase distribution of these PURs (Figure S8). In the AFM-phase images of PTMG1000-PUR and PCL1000-PUR, there were several bright and dark regions considered as hard and soft phase, respectively. But, in the AFM-phase images of PPG1000-PUR and PC1000-PUR, the bright and dark regions were hardly recognized. Moreover, unlike aromatic PUR with clear nanoscale ribbon morphology [19,49], these PURs had relatively indistinct microphase morphology. For quantitative insight into the degree of hard and soft segment microphase morphology, the mean interdomain spacings (d) of all these four PURs are calculated from approximately 14 nm through to absolute intensity SAXS in Table 2 and Figure S9. These results indicated that the macrodiol segments could not play an important role in phase separation in the PURs.

### 3.4. Mechanical Property Analysis

Surface hardness and tensile tests were performed to evaluate the mechanical properties of these PURs. Based on Shore A values of material surface, the hardness of the PURs from low to high is as follows: PTMG1000-PUR < PCL1000-PUR < PC1000-PUR < PPG1000-PUR. The tensile test was conducted using a universal tensile machine at a draw rate of 100 mm/min. Young’s modulus, ultimate tensile strength, elongation at break, and toughness are summarized in Table 3 and Figure S10. The ultimate tensile strength of these PURs from low to high is as follows: PTMG1000-PUR < PCL1000-PUR < PPG1000-PUR < PC1000-PUR, and the ultimate tensile strength and toughness of PC1000-PUR achieved 12.01 MPa and 44.27 MJ/m^3^, respectively, which were both much higher than the other three PURs. As a result, it implied that material mechanical properties were dependent on the soft segment.

**Figure 4:**
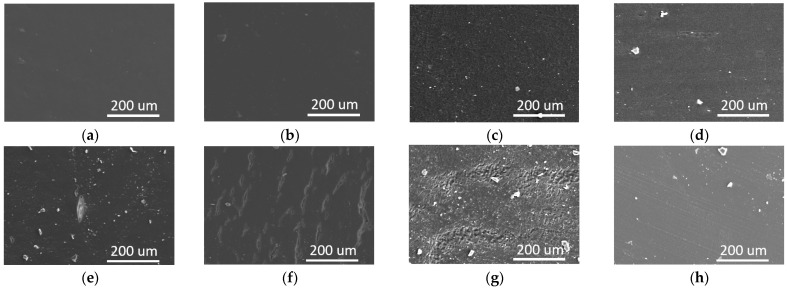
SEM images of the PURs: (**a**) PPG1000-PUR, (**b**) PTMG1000-PUR, (**c**) PCL1000-PUR, (**d**) PC1000-PUR before tensile testing, (**e**) PPG1000-PUR, (**f**) PTMG1000-PUR, (**g**) PCL1000-PUR, (**h**) PC1000-PUR after tensile testing.

In order to further investigate the effect of the soft phase in the PUR on its mechanical properties, the scanning electron microscope (SEM) images of these PURs before and after the tensile test are compared (Figure 4). Before the tensile test, the surface morphology of the four PURs were flat and smooth and no obvious crack was observed, as expected. However, the sample surface morphology of the four PURs became different after the same tensile test.

**Figure 5:**
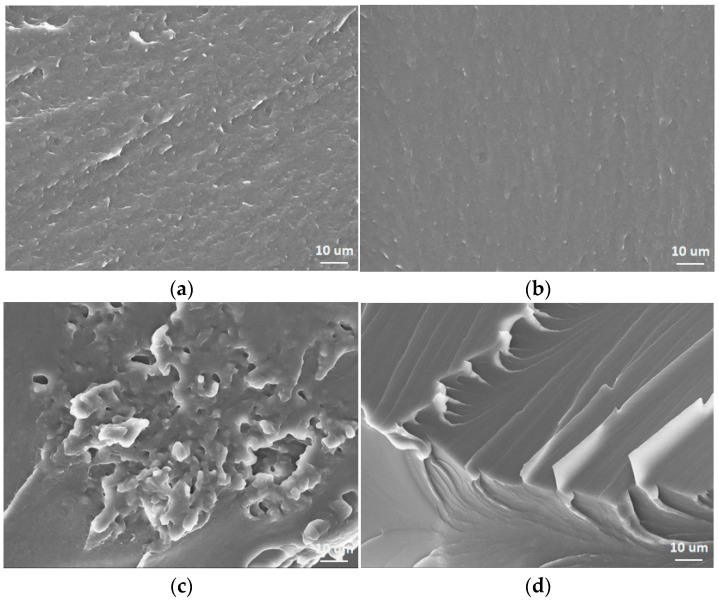
SEM images of tensile fracture surfaces of the four PURs: (**a**) PPG1000-PUR, (**b**) PTMG1000-PUR, (**c**) PCL1000-PUR, (**d**) PC1000-PUR.

Although PPG1000-PUR had a few cracks about 100 μm long on the surface, the entire surface was still kept flat and smooth. There were lots of similar cracks on the surface of PTMG1000-PUR, and many cracks were attached together to form longer cracks on the surface. Compared to PTMG1000-PUR, PCL1000-PUR showed more and smaller cracks to form a crazing zone on the surface. Interestingly, no crack was observed on the surface of PC1000-PUR. In other words, the rest parts of PC1000-PUR still kept good integrality, and the material had even been broken under tensile force. As above, the results suggested that relatively homogeneous microphase morphology was profitable to improve the mechanical properties of the PURs. The tensile fracture surfaces of four types of PUR materials were precisely analyzed (Figure 5). The tensile fracture surface of PC1000-PUR exhibited a significant amount of feather structures, indicating a relatively typical ductile fracture, which can absorb a large amount of energy. Similarly, the tensile fracture surfaces of PPG1000-PUR and PTMG1000-PUR materials also showed clear feather structures, while the feather structures on the tensile fracture surface of PTMG1000-PUR were smaller in size. In contrast, no feather structures were observed on the fracture surface of the PCL1000-PUR material. Instead, there were numerous clusters of particles approximately 5 um in size. These particles are likely the phase-separated products resulting from the crystallization of PCL1000 macrodiol, and these distinct particle clusters reduced the tensile performance of the material.

### 3.5. Dynamic Property Analyses

**Figure 6:**
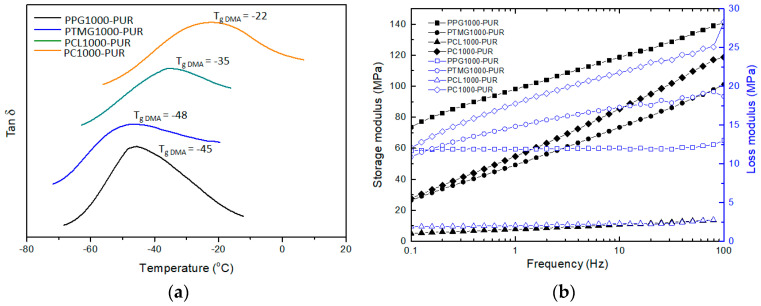
Dynamic properties of the PURs using DMA: (**a**) loss factors depending on temperature, (**b**) storage modulus and loss modulus depending on frequency.

The dynamic properties of the four PURs have been investigated using DMA in strain control. Figure 6a showed the temperature dependence of the loss factor (tan δ) of these PURs at 1 Hz. The peak temperatures marked as the T_g DMA_ were extracted in the tan δ curves. Compared to the above T_g DSC_ from DSC curves (Figure S4), all T_g DMA_ of the four PURs were higher, especially the T_g DMA_ of PC1000-PUR, which was up to 34 °C higher than its T_g DSC_. The frequency sweep dynamic properties of these PURs are showed in Figure 6b at 25 °C. The storage modulus of the four PURs increased obviously, and the storage modulus of PC1000-PUR gained the most improvement by 90 MPa from 0.1 Hz to 100 Hz when the test frequency increased. It suggested that PC1000-PUR could have better mechanical properties at a high frequency. Moreover, the loss modulus of PTMG1000-PUR and PC1000-PUR increased gradually when the test frequency increased, and the loss modulus of PPG1000-PUR and PCL1000-PUR was not sensitive to the change in frequency. Based on the DMA data, PC1000-PUR could be better at absorbing the energy of high-frequency waves.

### 3.6. Fraction-Free Volume Analysis

In an effort to gain further insight into the effect of the soft segment on energy absorption at the molecular level, the molecular models of the four PURs were built in Materials Studio. The fraction-free volume (FFV) analysis reflected the chain packing efficiency of polymer molecules, and a lower FFV indicated tighter atomic packing [50]. The FFV calculations for the four PURs are shown in Table 4. It is worth mentioning that the FFV of the four polymers was calculated by the following equation (Equation (1)) [44]. PTMG1000-PUR had the highest FFV among the four materials, indicating a low dynamic glass transition temperature of the material, which was consistent with the above DMA data:

(1) $$FFV=\frac{V-1.3V_{W}}{V}$$

where V_w_ was the specific Van der Waals volume of molecules. Also, V was the volume of the molecular cell.

### 3.7. Binding Energy Analysis

The binding energy (E_bind_), the negative value of the interaction energy (E_inter_) of macrodiol and resin segments of PURs, could be evaluated using the interaction strength between macrodiol and resin segments. In this paper, E_inter_ was expressed as the difference between the total energy of the system and the energy of each component. Through MD simulation, the total energy of the system (E_total_), the energy of macrodiol segments(E_MS_), and the energy of resin segments (E_RS_) could be calculated, respectively, and E_inter_ could be calculated through the following formula (Equation (2)).

E_bind_ = −E_inter_ = − ( E_total_ − E_ss_ − E_Hs_) (2)

**Figure 7:**
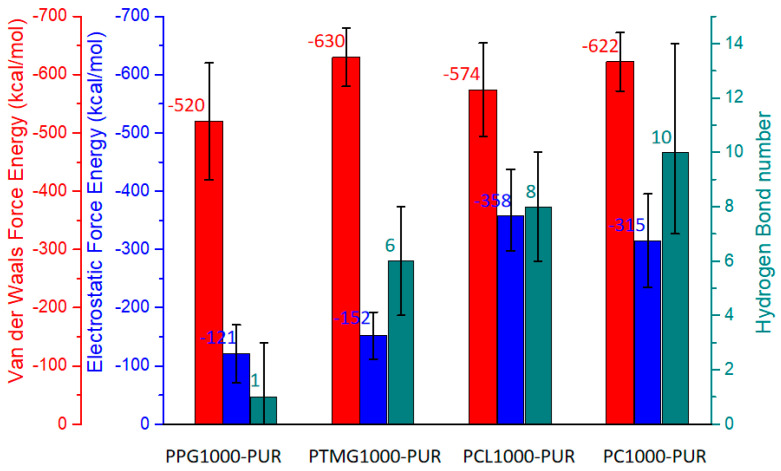
Van der Waals force energy, electrostatic force energy, and H-Bond number on the interface of macrodiol segments and resin segments of the PURs.

According to the E_bind_ sequence of the four PURs, PPG1000-PUR (656 kcal/mol) < PTMG1000-PUR (798 kcal/mol) < PCL1000-PUR (938 kcal/mol) < PC1000-PUR (942 kcal/mol), there were relatively strong interaction forces between the macrodiol and resin segments of polyester-type PC1000-PUR and PCL1000-PUR because of polar C=O groups of PC1000 and PCL1000. In the Compass force field, the non-bond forces were divided into Van der Waals force and electrostatic force. For the non-bond forces between macrodiol and resin segments of the four PURs, the Van der Waals force was the main interaction force, because the Van der Waals force energy far outweighed the electrostatic force energy (Figure 7). Furthermore, the number of H-Bonds was also an important indicator. The H-Bond number between the macrodiol and resin segments of PCL1000-PUR and PC1000-PUR were also more than that of the other two polyether-type PURs.

### 3.8. Mean Square Radius of Gyration Analysis

**Figure 8:**
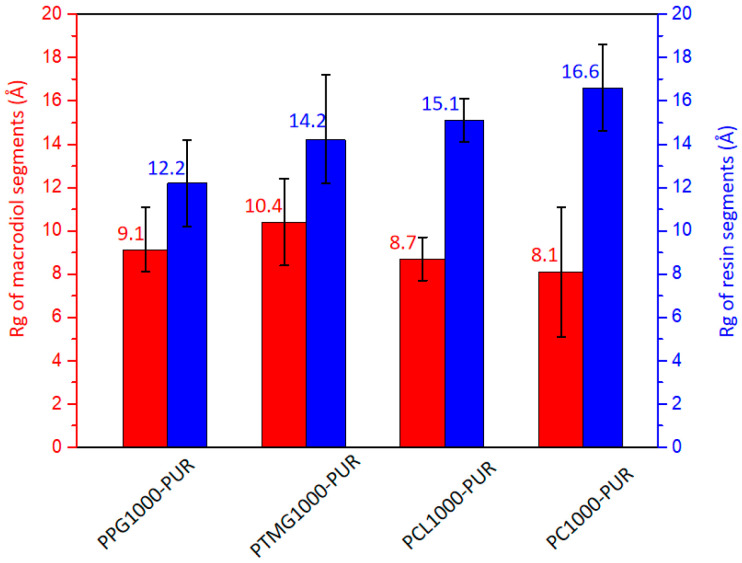
Mean square radius of the gyration of the macrodiol segments and resin segments in the PURs.

The mean square radius of gyration (R_g_) was an important size parameter of polymer chains. For the same molecular weight, the larger R_g_ represented the looser polymer chains and better polymer chain compatibility. In order to study the distribution of macrodiol and resin segments of PURs, R_g_ of the macrodiol and resin segments of the PURs was calculated separately with the Analysis module in the MD simulation (Figure 8). The mean square R_g_ is obtained by statistically averaging the R_g_ of the macrodiol or resin segments from the four PUR chains. The R_g_s of macrodiol segments of PURs were relatively similar in the range of 8.1 to 10.4 Å because of the similar molecular weight of macrodiol segments. However, there was a noticeable difference for the R_g_s of resin segments of PURs, especially PC1000-PUR, which showed the biggest R_g_ of resin segments. This was probably because of an increase in the intermolecular force from PC1000 as macrodiol segments stretch the resin segment chains, resulting in an increase in the mean square rotation of the hard segment chains. The compatibility between these two segments becomes better and better.

### 3.9. Energy Absorption Mechanism

In order to investigate the energy absorption of the four PURs at the molecular level, a series of simple shear deformation tests were run under different shear rate conditions through MD simulation. In this paper, the total energy of the material system mainly included the internal energy (temperature) and the potential energy (E_potential_), in which the potential energy was the sum of the bond energy (E_bond_) and non-bond energy (E_non-bond_) of polymers. An energy minimization procedure was used in these tests to observe the effect of shear rate on the changes in temperature, energy, and H-Bond number in the four PURs.

**Figure 9:**
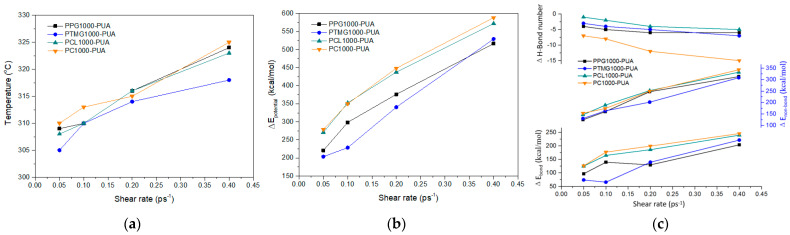
Changes in the PURs depending on shear rate: (**a**) temperature trends, (**b**) ΔE_potential_ trends, (**c**) ΔE_bond_, ΔE_non-bond_, and ΔH-Bond.

**Figure 10:**
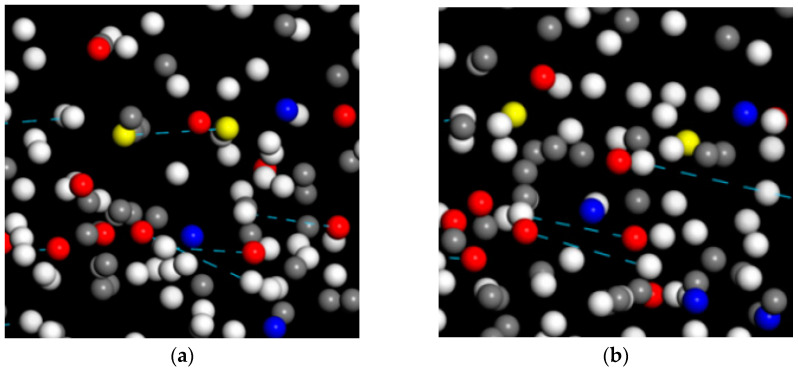
A zoomed-in view of the PC1000-PUR model (**a**) before and (**b**) after shear deformation. The H-bonds were depicted as dashed lines and the yellow balls were object atoms.

With shear rate increased, the temperature and E_potential_ of the four PURs gradually rose, indicating that one part of the external energy from simple shear deformation could be absorbed to convert into the internal energy of PURs, and the other part of the energy into E_potential_ (Figure 9). Among the four PURs, the system temperature of PTMG1000-PUR increased the least, probably because of the low atomic density in PTMG1000-PUR and low friction between the atoms, while PC1000-PUR had the highest system temperature change. With the increase in shear rate, the E_potential_ of the four PURs from high to low is as follows: PC1000-PUR > PCL1000-PUR > PTMG1000-PUR > PPG1000-PUR. Classifying the E_potential_ into E_bond_ and E_non-bond_, it was found that the change in E_non-bond_ was more substantial, accounting for about 60% of the E_potential_ change, and only 40% of the E_potential_ change for E_bond_ (Figure 9c). Furthermore, the number of H-Bonds changed very significantly in the E_non-bond_. With the increase in shear rate, the number of H-Bonds decreased rapidly, especially for PC1000-PUR, with a decrease of 15. According to the above simulation calculation, under the dynamic load conditions, the external energy absorption was mainly converted into the E_non-bond_ of PURs, and the H-Bond deformation in the E_non-bond_ was the main index of PUR energy absorption (Figure 10).

Based on the combination of experimental results and simulation data, an effective quantification of the performance and energy absorption mechanism of PURs with different macrodiol structural units at the molecular level was provided. Tensile testing revealed that PC1000-PUR exhibited the highest tensile strength and toughness among the four PURs, attributed to the extensive formation of ordered hydrogen bonds and homogeneous phase distribution. PTMG1000 and PCL1000 showed relatively lower mechanical strength and more surface cracks due to the crystallization of their macrodiol segments. In terms of dynamic properties, PC1000-PUR displayed higher storage modulus and loss modulus with increasing frequency, and its glass transition temperature (T_g DMA_) was the highest, reaching −22 °C at 1 Hz under dynamic mechanical analysis. MD simulations indicated that PC1000 diols exhibited stronger interaction (approximately 942 kcal/mol) with the resin segments at the molecular level, suggesting their significant contribution to the mechanical properties of the material. The energy absorption mechanism of the PURs was primarily attributed to the change in non-bonded energy, accounting for approximately 60% of the total energy absorbed under dynamic loading conditions. H-Bond deformation was identified as an important factor in energy absorption.

## 4. Conclusions

The mechanical and dynamic properties of four polyurethane elastomers (PURs) with different macrodiol segments were studied. PC1000-PUR exhibited the highest tensile strength and toughness due to ordered H-bond formation and homogeneous phase distribution. PTMG1000 and PCL1000 showed lower mechanical strength and more surface cracks. PC1000-PUR had higher storage and loss modulus, with the highest T_g DMA_. MD simulations revealed a strong interaction between PC1000 diols and resin segments. Energy absorption in PURs was primarily attributed to non-bonded energy change, with H-Bond deformation playing a crucial role. This study provides insights for designing PURs with optimal energy absorption.

## Figures and Tables

**Table 1:** Density of PURs determined through experiment and MD simulation.

Sample Name	PPG1000-PUR	PTMGG1000-PUR	PCL1000-PUR	PC1000-PUR
Density_Exp_ (g/cm^3^)	1.021	1.007	1.051	1.053
Density_MD_ (g/cm^3^)	1.041	1.019	1.045	1.055
Error (%)	1.9	1.2	0.6	0.2

**Table 2:** Polymerization results and polymer information.

Sample Name	*M_n_^a^*	MWD *^a^*	*M_n_^b^*	MWD *^b^*	T_g__DSC_ *^c^*	T_g__DMA_ *^d^*	T_d_ *^e^*	d *^f^*
	(×10^3^ g/mol)		(×10^3^ g/mol)		(°C)	(°C)	(°C)	(nm)
PPG1000-PUR	10.87	1.62	10.45	1.33	−60	−45	243	14.9
PTMG1000-PUR	10.19	1.73	9.51	1.42	−65	−48	292	14.8
PCL1000-PUR	9.33	1.77	7.97	1.48	−57	−35	282	14.5
PC1000-PUR	11.72	1.84	10.81	1.55	−56	−22	270	14.8

*^a^* determined by GPC. *^b^* determined by SLS. *^c^* determined by DSC. *^d^* determined by DMA. *^e^* determined by TGA. *^f^* calculated from the peak maxima (q_max_) of SAXS curves, d = 2π/q_max_.

**Table 3:** Mechanical properties of the PURs.

Sample Name	Surface Hardness	Young’s Modulus (MPa)	Tensile Strength (MPa)	Elongation at Break (%)	Toughness (MJ/m^3^)
PPG1000-PUR	88	80	5.19	182	8.39
PTMG1000-PUR	78	20	2.71	262	5.91
PCL1000-PUR	82	21	2.85	248	6.31
PC1000-PUR	87	29	12.01	688	44.27

**Table 4:** Fraction-free volumes of the PURs.

Sample Name	PPG1000-PUR	PTMG1000-PUR	PCL1000-PUR	PC1000-PUR
FFV	18.6	19.8	18.8	18.6

## Data Availability

The original contributions presented in the study are included in the article/supplementary material, further inquiries can be directed to the corresponding author.

## Supplementary Materials

The following supporting information can be downloaded at: https://www.mdpi.com/article/10.3390/polym17010107/s1, Figure S1. SEM of the four PUR cross sections. Figure S2. GPC curves of the PURs. Figure S3. DSC curves of the PURs for the first heating scans. Figure S4. DSC curves of the PURs for the second heating scans. Figure S5. TGA curves of the PURs. Figure S6. WAXD profiles of the PURs. Figure S7. AFM images of the PURs. Figure S8. AFM images of the PURs: (a) PPG1000-PUR, (b) PTMG1000-PUR, (c) PCL1000-PUR, (d) PC1000-PUR. Figure S9. SAXS profiles of the PURs. Figure S10. Stress-strain curves of the PURs.
