# Supplementary material for: Dynamic Mechanical Properties and Energy Absorption Capabilities of Polyureas Through Experiments and Molecular Dynamic Simulation
Source: Polymers (Basel). 2025 Jan 2;17(1):107. doi: 10.3390/polym17010107 (PMC11722925; doi:10.3390/polym17010107)
Supplement: Supplementary file 1 [file polymers-17-00107-s001.zip › polymers-3376131-supplementary.pdf]

# Supporting information

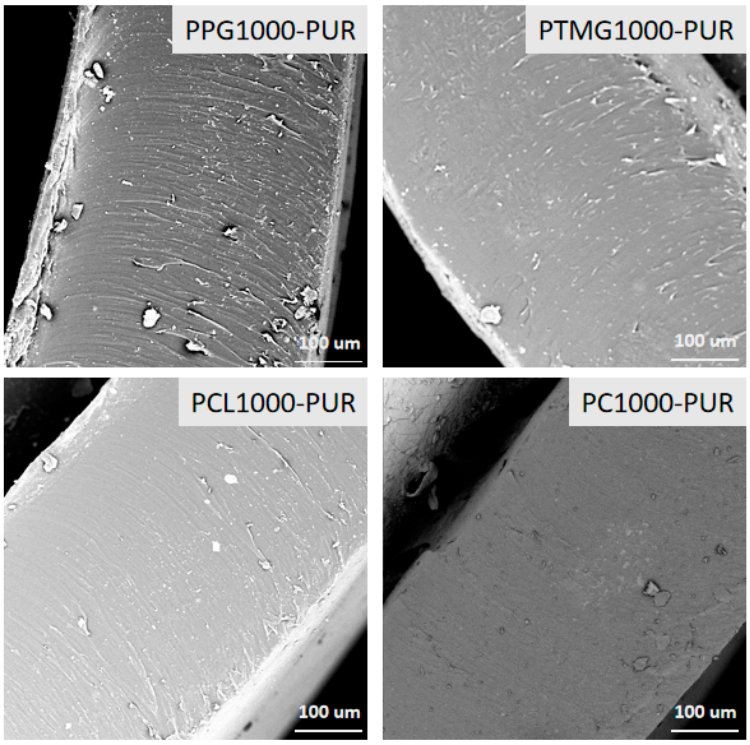


**Figure S1.** SEM of the four PUR cross sections.

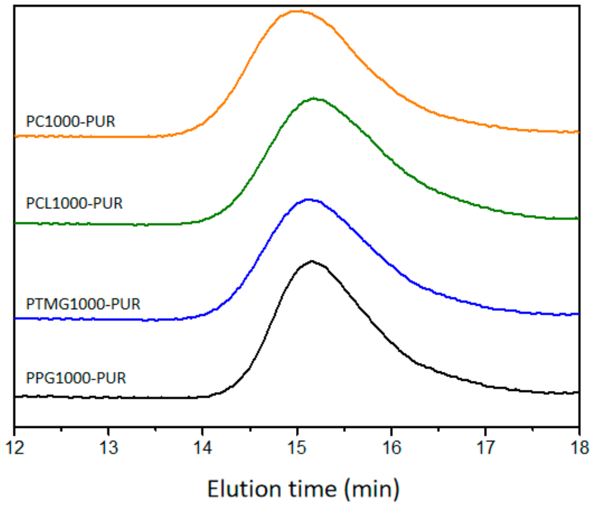


**Figure S2.** GPC curves of the PURs.

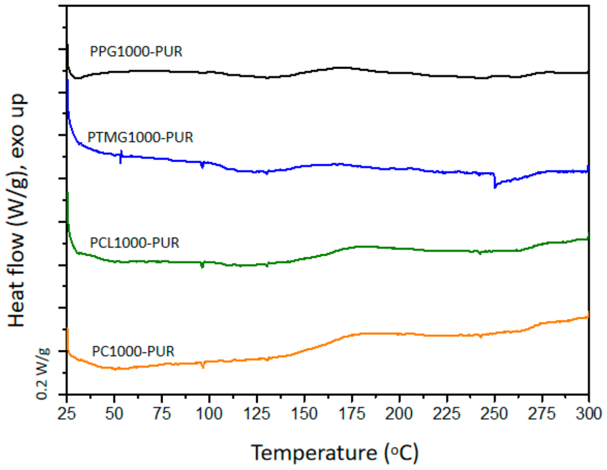


**Figure S3.** DSC curves of the PURs for the first heating scans.

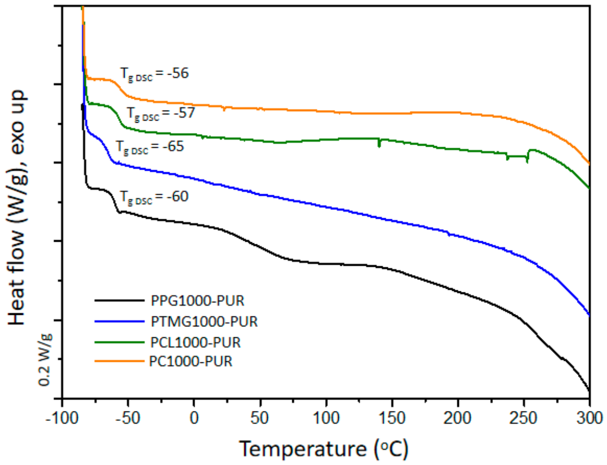


**Figure S4.** DSC curves of the PURs for the second heating scans.

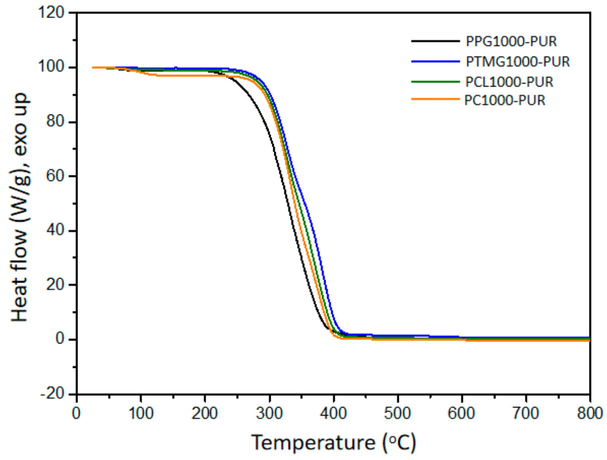


**Figure S5.** TGA curves of the PURs.

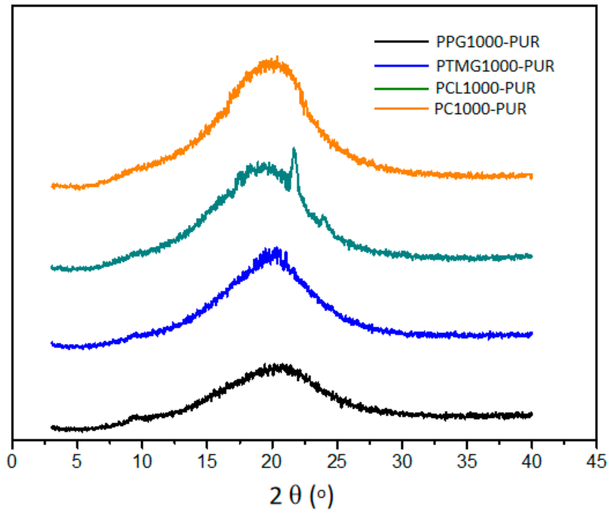


**Figure S6.** WAXD profiles of the PURs.

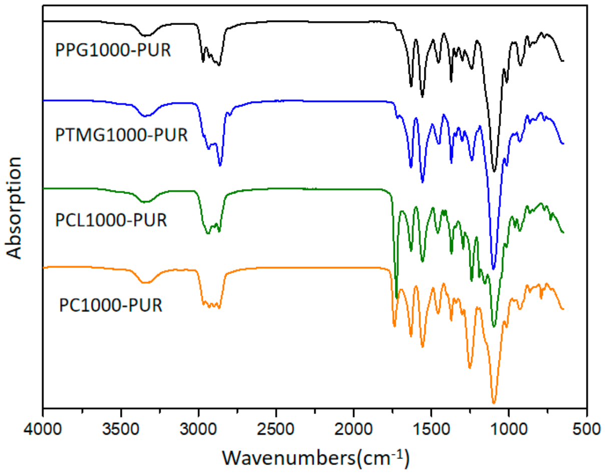


**Figure S7.** AFM images of the PURs.

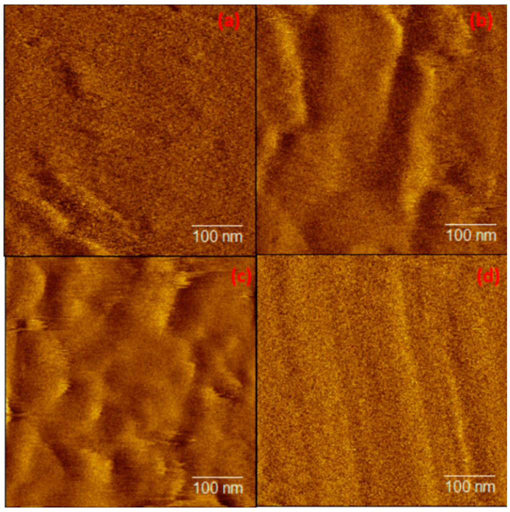


**Figure S8.** AFM images of the PURs: (a) PPG1000-PUR, (b) PTMG1000-PUR, (c) PCL1000-PUR, (d) PC1000-PUR.

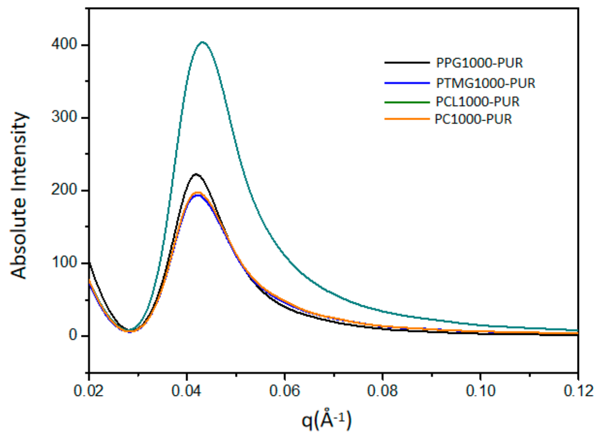


**Figure S9.** SAXS profiles of the PURs.

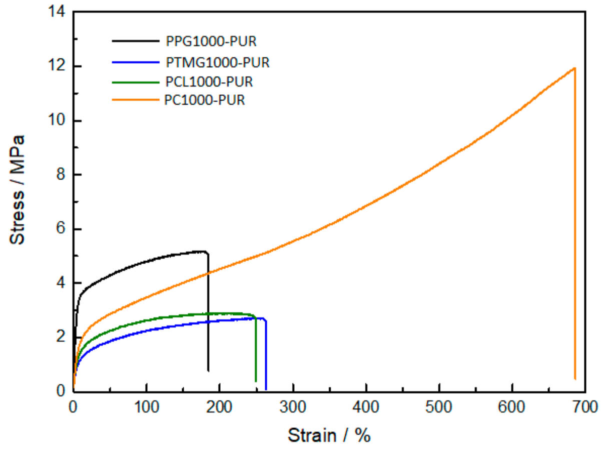


**Figure S10.** Stress-strain curves of the PURs.
